# Nerve as a Barometer

**Published:** 1884-07

**Authors:** 


### Nerve as a Barometer.

The fact of a relation between neuralgic pain and meteorological conditions is generally understood. The coming change of weather is responsible for many of the aches and pains the nervous system is heir to. Depression of spirits and rheumatic pains are generally associated with a falling barometer and storm-brewing conditions. Unusually severe neuralgic attacks coincide with unusually intense storm development. To establish in his own case the relation of pain and weather, Capt. Catlin, of the U. S. Army, made a regular and detailed record, in connection with the variations of the weather, of the variations of the neuralgic pains with which he is afflicted. Captain Catlin's foot was crushed by a shot in 1864, and it was necessary to amputate his leg below the knee. He continued to experience sensations of pain, as if in the lost member; these sensations being greater or less according to the atmospheric disturbance. He found the winter months to be the greatest pain-producers. Arranged in months, March naturally, as one would suppose, took the lead. Then came in order January, November, December, May, February, April, August, October, September, July, and June. He traced the average distance of the storm-center at the beginning of the pain attack by investigating sixty well-defined storms in a period of ten consecutive months. This average distance was six hundred and eighty miles, the range being from two to twelve hundred miles. It is curious and interesting information that at the beginning of our neuralgic attacks the storm development causing them may be a thousand miles away.—*Cincinnati Commercial Gazette.*

